# Axonal Non-segregation of the Vesicular Glutamate Transporter VGLUT3 Within Serotonergic Projections in the Mouse Forebrain

**DOI:** 10.3389/fncel.2019.00193

**Published:** 2019-05-10

**Authors:** Arnauld Belmer, Kate Beecher, Angela Jacques, Omkar L. Patkar, Florian Sicherre, Selena E. Bartlett

**Affiliations:** ^1^Translational Research Institute, Institute of Health and Biomedical Innovation, Queensland University of Technology, Brisbane, QLD, Australia; ^2^QIMR Berghofer Medical Research Institute, Institute of Health and Biomedical Innovation, Queensland University of Technology, Brisbane, QLD, Australia; ^3^Biologie Integrative et Physiologie, Université Pierre et Marie Curie, Paris, France

**Keywords:** serotonin, serotonin transporter, SERT, VGLUT3, 5-HT varicosity

## Abstract

A subpopulation of raphe 5-HT neurons expresses the vesicular glutamate transporter VGLUT3 with the co-release of glutamate and serotonin proposed to play a pivotal role in encoding reward- and anxiety-related behaviors. Serotonin axons are identifiable by immunolabeling of either serotonin (5-HT) or the plasma membrane 5-HT transporter (SERT), with SERT labeling demonstrated to be only partially overlapping with 5-HT staining. Studies investigating the colocalization or segregation of VGLUT3 within SERT or 5-HT immunolabeled boutons have led to inconsistent results. Therefore, we combined immunohistochemistry, high resolution confocal imaging, and 3D-reconstruction techniques to map and quantify the distribution of VGLUT3 immunoreactive boutons within 5-HT vs. SERT-positive axons in various regions of the mouse forebrain, including the prefrontal cortex, nucleus accumbens core and shell, bed nucleus of the stria terminalis, dorsal striatum, lateral septum, basolateral and central amygdala, and hippocampus. Our results demonstrate that about 90% of 5-HT boutons are colocalized with SERT in almost all the brain regions studied, which therefore reveals that VGLUT3 and SERT do not segregate. However, in the posterior part of the NAC shell, we confirmed the presence of a subtype of 5-HT immunoreactive axons that lack the SERT. Interestingly, about 90% of the 5-HT/VGLUT3 boutons were labeled for the SERT in this region, suggesting that VGLUT3 is preferentially located in SERT immunoreactive 5-HT boutons. This work demonstrates that VGLUT3 and SERT cannot be used as specific markers to classify the different subtypes of 5-HT axons.

## Introduction

Intensive efforts have long been made to understand the complexity of the serotonin (5-HT) system and to identify specific markers for serotonin neuron diversity. Although it is becoming evident that raphe serotonin neurons are morphologically, functionally, and molecularly heterogeneous (Calizo et al., 2011; Kiyasova et al., 2011, 2013; Gaspar and Lillesaar, 2012; Fernandez et al., 2016), the diversity of serotonergic axonal projections to the forebrain is not completely understood.

Pioneer electron or light microscopy and anterograde tracing studies have revealed the existence of 5-HT axon terminals with different sizes, shapes, contents of their small vesicles, and the presence or absence of dense-core vesicles [for review see Descarries et al. (2010)]. In rats, two types of axons were reported, with axons originating from the dorsal raphe showing fine beaded or fusiform varicosities separated by smooth axon segments of variable length (type D), while axons originating from the median raphe displayed large spherical varicosities with fine and smooth inter-varicosity segments (type M) (Kosofsky and Molliver, 1987). In primates, two types of axons were also described, with sparse, small, ovoid, or large, spheroidal varicosities (Hornung et al., 1990). However, it is likely that this axonal morphology classification cannot longer be considered as valid criteria for distinguishing the cellular origin or the chemical identity of the 5-HT neurons. Indeed a chemically defined 5-HT neuron can send several types of axonal projections with different morphologies to different brain regions (Gagnon and Parent, 2014). Hence, research has been rather devoted to studying the molecular or physiological diversity of 5-HT neurons, identifying various 5-HT neuronal subtypes that differentially express the 5-HT1A autoreceptor (Sotelo et al., 1990; Kirby et al., 2003; Bonnavion et al., 2010; Kiyasova et al., 2013; Fernandez et al., 2016), substance P/neurokinin receptor 1 (NK1; Lacoste et al., 2006), galanin and its receptor (Xu and Hökfelt, 1997; Larm et al., 2003), neuronal nitric oxide synthase (nNOS; Xu and Hökfelt, 1997), gamma-aminobutyric acid (GABA)-synthesizing enzyme glutamic acid decarboxylase (GAD; Fu et al., 2010), alpha7 nicotinic receptor (Aznar et al., 2005), MET receptor tyrosine kinase (Kast et al., 2017) or display different pharmacological and electrophysiological properties (Kirby et al., 2003; Hajós et al., 2007; Calizo et al., 2011). This heterogeneity appears to be target-specific (Fernandez et al., 2016; Prouty et al., 2017) and could therefore be used to establish a specific anatomy/function cartography of raphe serotonin sub-systems (Ren et al., 2018).

In addition, a subpopulation of dorsal and median raphe 5-HT neurons was found to co-express transcripts of the vesicular glutamate transporter type 3 (VGLUT3; Gras et al., 2002; Hioki et al., 2010), suggesting that 5-HT and glutamate could be stored in the same vesicles and co-released. VGLUT3 protein was also reported to be located in the some 5-HT immunoreactive axonal varicosities in the forebrain (Schäfer et al., 2002; Mintz and Scott, 2006), including the granular cell layer of the olfactory bulb, cerebral cortex, central amygdaloid nuclei, hippocampal CA3 field, dorsolateral septum, and supra-ependymal plexus of the third ventricle (Shutoh et al., 2008). A classification of two serotonergic axons subtypes depending on the presence or absence of VGLUT3 was therefore proposed (Shutoh et al., 2008). It is likely that the co-expression of VGLUT3 and the vesicular monoamine transporter 2 (Vmat2) in serotonin terminals (Schäfer et al., 2002) synergizes the filling of 5-HT and glutamate in the same synaptic vesicles (Amilhon et al., 2010), with 5-HT/glutamate cotransmission proposed to play a pivotal role in the control of reward- and emotion-related neural circuitry (Liu et al., 2014; Sengupta et al., 2017) and their plasticity/adaptability during development or pathological processes (Gagnon and Parent, 2014).

However, some discrepancies have emerged from the aforementioned studies, regarding the total or sparse colocalization of the SERT in 5-HT axon varicosities. While Gagnon and Parent (2014) observed that all 5-HT axon varicosities contain the SERT in rats, two studies have reported a very sparse colocalization of SERT and 5-HT immunolabeling in mice, with VGLUT3 and SERT mostly segregated within 5-HT varicosities (Amilhon et al., 2010; Voisin et al., 2016), especially in the prefrontal cortex, HIP, dorsal striatum, and LS. This data suggests that different subtypes of 5-HT axonal varicosities (SERT^-^/VGLUT3^+^ or SERT^+^/VGLUT3^-^) may coexist in the mouse forebrain.

In the present study, we therefore investigated the immunohistological distribution of SERT and VGLUT3 within 5-HT axon varicosities in various regions of the mouse forebrain, including the prefrontal cortex, NAc core and NAc shell, BNST, dorsal striatum, LS, BLA and CeA, and HIP. For this, we imaged ±1.55 × 10^8^ μm^3^ of tissue and 3D-reconstructed ±10^6^ 5-HT varicosities to determine the volumetric density and the proportion of varicosities co-labeled with SERT and/or VGLUT3, in each brain region. We found that the great majority (≈90%) of 5-HT varicosities express the SERT in every brain regions analyzed except the posterior shell of the NAc (50%). We herein report that VGLUT3 is preferentially located in SERT^+^ 5-HT varicosities. Our results demonstrate that VGLUT3 and SERT do not particularly segregate in 5-HT axonal varicosities of the mouse forebrain.

## Materials and Methods

### Animals

Six 8–10-week-old C57Bl6 mice (3 males, 3 females) were housed in standard ventilated cages in climate-controlled rooms. Food, water, and environmental enrichment were available *ad libitum*. This study was carried out in accordance with the recommendations of National Health and Medical Research Council (NHMRC) guidelines to promote the well-being of animals used for scientific purposes and the Australian code for the care and use of animals for scientific purposes. The protocol was approved by the Queensland University of Technology Animal Ethics Committee and the University of Queensland Animal Ethics Committee.

### Histology

Mice were deeply anesthetized with 100 mg/kg of pentobarbital (Lethobarb, Virbac, Australia) and transcardially perfused with 4% paraformaldehyde (PFA) prior to decapitation. Brains were harvested and post-fixed overnight at 4°C. Forty-micron thick coronal vibratome sections were collected and incubated overnight in blocking solution [2% normal goat serum, 0.3% Triton, and 0.05% Tween 20 in 0.1 M phosphate-buffer saline (PBS)].

### Immunohistochemistry

Sections containing the prelimbic cortex (Bregma: +2.46 ± 0.3 mm), the NAC (Bregma: +1.42 ± 0.2 mm), the posterior NAC, the dorsal striatum and the LS (Bregma: +1.00 ± 0.2 mm), the BNST (Bregma: -0.22 ± 0.3 mm), the hippocampus (Bregma: -1–70 ± 0.3 mm), or the amygdala (Bregma: -1.40 ± 0.2 mm) were incubated with primary antibodies diluted in the blocking solution: rat anti-5-HT (Millipore #MAB352, 1:100) for 48 h at room temperature followed by rabbit anti-SERT (Millipore #PC177L, 1:1000) and the KO-validated (Fasano et al., 2017) guinea-pig anti-VGLUT3 (Synaptic System #135204), at 1:500 dilution [as per supplier’s recommendations and other studies (Puighermanal et al., 2017)], overnight at 4 degrees. After three washes in the blocking solution, the slices were incubated for 4 h at room temperature with secondary antibodies diluted in the blocking solution: goat anti-rabbit-Alexa 488, goat anti-guinea pig-Alexa 647 (Thermofisher Scientific, #A11034 and #A21450, 1:500) and goat anti-rat biotinylated (Jackson Laboratory # 112-065-003, 1:200). After three washes in the blocking solution, slices were incubated for 30 min in Streptavidin-Cy3 (Thermofisher Scientific #438315, 1:1000), washed three times in PBS, and mounted in Prolong Gold antifade mountant (Thermofisher Scientific, #P36934).

### Imaging and Analysis

**FIGURE 1:**
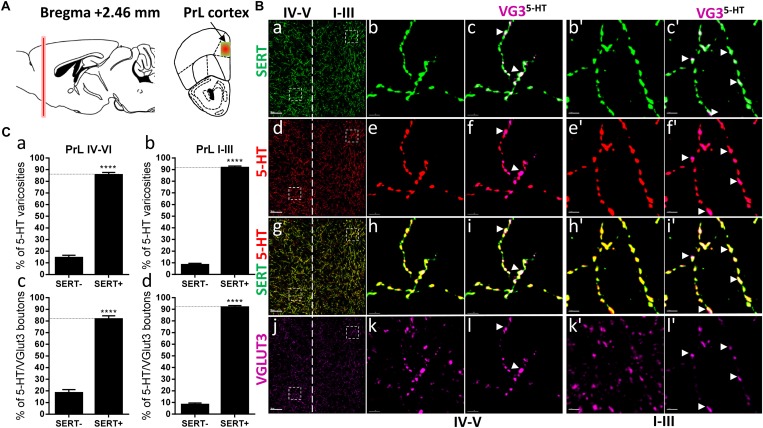
Distribution of VGLUT3^+^ boutons within 5-HT^+^ and SERT^+^ axons in the prelimbic cortex. **(A)** Schematic drawing showing the location of the acquired micrographs. Prelimbic (PrL) cortex mosaic images of layer I–V were acquired at bregma +2.46 ± 0.2 mm (red vertical line) in the dorsal half of the medial cortex (yellow/red square). **(B)** Micrograph showing the distribution of the SERT (green, **a–c,b’,c’**), 5-HT (red, **d–f; e’,f’**), SERT (green) + 5-HT (red) **(g–i, h’,i’)**, and VGLUT3 (magenta, **j–l, k’,l’**). Left panel shows lower magnification (**a,d,g,j**). Scale bar: 50 μm. Middle panel shows higher magnification of the white dash box for layers IV–V (**b,c,e,f,h,i,k,l**) and right panel shows higher magnification white dash box for layers I–III **(b’,c’,e’,f’,h’,i’,k’,l’)**. Scale bar: 2 μm. The panel labeled VG3^-5-HT^ shows the VGLUT3 boutons co-labeled with SERT **(c,c’)**, 5-HT **(f,f’)**, and SERT^+^5-HT **(i,i’)**. VGLUT3 puncta located within 5-HT varicosities were isolated using Imaris **(l,l’)**. Arrowheads show 5-HT varicosities collocated with SERT and VGLUT3 **(c,c’,f,f’,i,i’,l,l’)**. **(C: a,b)** Quantification of the proportion of 5-HT varicosities not co-labeled (SERT^-^) and co-labeled (SERT^+^) with SERT in the upper **(I–III)** or deeper **(IV,V)** layers of the PrL, showing a great majority of 5-HT varicosities co-labeled with SERT. **(c,d)** Quantification of the proportion of 5-HT^+^/VGLUT3^+^ not co-labeled (SERT^-^) and co-labeled (SERT^+^) with SERT in the upper **(I–III)** or deeper **(IV,V)** layers of the PrL, showing a great majority of 5-HT^+^/VGLUT3^+^ varicosities co-labeled with SERT (*t*-test, ^∗∗∗∗^: *p* < 0.0001).

**FIGURE 2:**
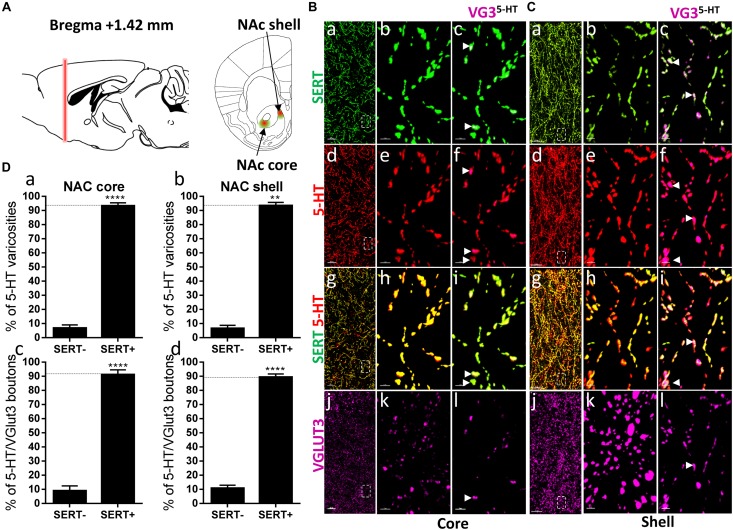
Distribution of VGLUT3^+^ boutons within 5-HT^+^ and SERT^+^ axons in the nucleus accumbens. **(A)** Schematic drawing showing the location of the acquired micrographs. Nucleus accumbens (NAc) mosaic images were acquired at bregma +1.42 ± 0.2 mm (red vertical line) in the dorsomedial shell and the core (ventrolateral to the anterior commissure) of the nucleus accumbens (green/red areas). **(B)** Micrograph showing the distribution of the SERT (green, **a–c**), 5-HT (red, **d–f**), SERT (green) + 5-HT (red) (**g–i**), and VGLUT3 (magenta, **j–l**) in the NAc core. Left panel **(a,d,g,j)** shows lower magnification. Scale bar: 50 μm. Middle **(b,e,h,k)** and right **(c,f,i,l)** panels show higher magnification of the white dashed box in the left panel **(a,d,g,j)**. Scale bar: 2 μm. The right panel shows the VGLUT3 boutons co-labeled with SERT **(c)**, 5-HT **(f)**, and SERT + 5-HT **(i)**. VGLUT3 puncta located within 5-HT varicosities were isolated using Imaris **(l)**. Arrowheads show 5-HT varicosities collocated with SERT and VGLUT3 **(c,f,i,l)**. **(C)** Micrograph showing the distribution of the SERT (green, **a–c**), 5-HT (red, **d–f**), SERT (green) + 5-HT (red) (**g–i**), and VGLUT3 (magenta, **j–l**) in the Nac shell. Left panel **(a,d,g,j)** shows lower magnification. Scale bar: 50 μm. Middle **(b,e,h,k)** and right **(c,f,I,l)** panels show higher magnification of the white dashed box in the left panel **(a,d,g,j)**. Scale bar: 2 μm. The right panel shows the VGLUT3 boutons co-labeled with SERT **(c)**, 5-HT **(f)**, and SERT + 5-HT **(i)**. VGLUT3 puncta located within 5-HT varicosities were isolated using Imaris **(l)**. Arrowheads show 5-HT varicosities collocated with SERT and VGLUT3 **(c,f,i,l)**. **(D: a,b)** Quantification of the proportion of 5-HT varicosities not co-labeled (SERT^-^) and co-labeled (SERT^+^) with SERT in the NAc, showing a great majority of 5-HT varicosities co-labeled with SERT. **(c,d)** Quantification of the proportion of 5-HT^+^/VGLUT3^+^ not co-labeled (SERT–) and co-labeled (SERT^+^) with SERT in the NAc, showing a great majority of 5-HT^+^/VGLUT3^+^ varicosities co-labeled with SERT (*t*-test, ^∗∗^*p* < 0.01, ^∗∗∗∗^*p* < 0.0001).

**FIGURE 3:**
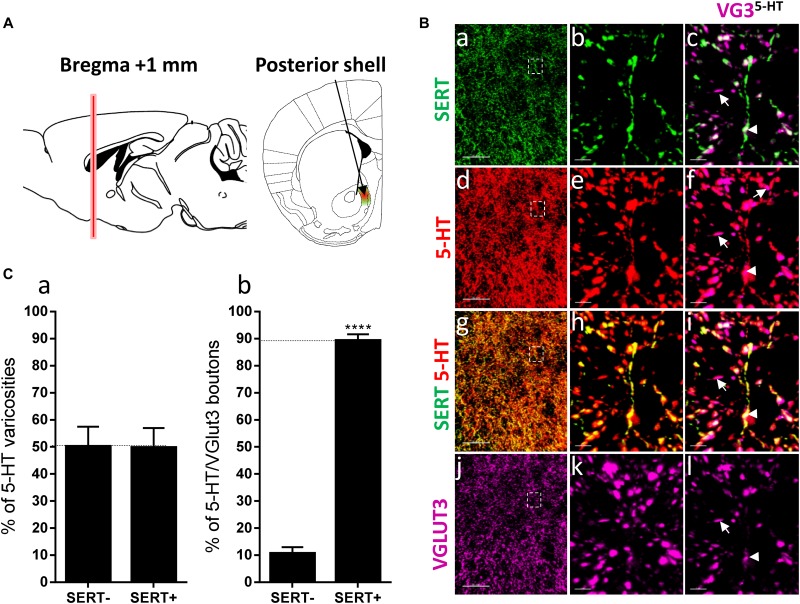
Distribution of VGLUT3^+^ boutons within 5-HT^+^ and SERT^+^ axons in the posterior shell of the nucleus accumbens. **(A)** Schematic drawing showing the location of the acquired micrographs. The posterior (caudal) shell of the nucleus accumbens (NAc) (green/red area) mosaic images were acquired at bregma +1 ± 0.2 mm (red vertical line). **(B)** Micrograph showing the distribution of the SERT (green, **a–c**), 5-HT (red, **d–f**), SERT (green) + 5-HT (red) (**g–i**), and VGLUT3 (magenta, **j–l**). Right panel (**a,d,g,j**) shows lower magnification. Scale bar: 50 μm. Middle (**b,e,h,k**) and right (**c,f,i,l**) panels show higher magnification of the white dashed box in the left panel **(a,d,g,j)**. Scale bar: 2 μm. The right panel **(c,f,i,l)** shows the VGLUT3 boutons co-labeled with SERT **(c)**, 5-HT **(f)** and SERT + 5-HT **(i)**. VGLUT3 puncta located within 5-HT varicosities were isolated using Imaris **(l)**. Arrowheads show 5-HT varicosities collocated with SERT and VGLUT3 **(c,f,i,l)**. **(C:a)** Quantification of the proportion of 5-HT varicosities not co-labeled (SERT^-^) and co-labeled (SERT^+^) with SERT in the posterior shell of the NAc, showing a similar proportion of 5-HT varicosities co-labeled with SERT. **(b)** Quantification of the proportion of 5-HT^+^/VGLUT3^+^ not co-labeled (SERT^-^) and co-labeled (SERT^+^) with SERT in the posterior shell of the NAc, showing a great majority of 5-HT^+^/VGLUT3^+^ varicosities co-labeled with SERT (*t*-test, ^∗∗∗∗^*p* < 0.0001).

**FIGURE 4:**
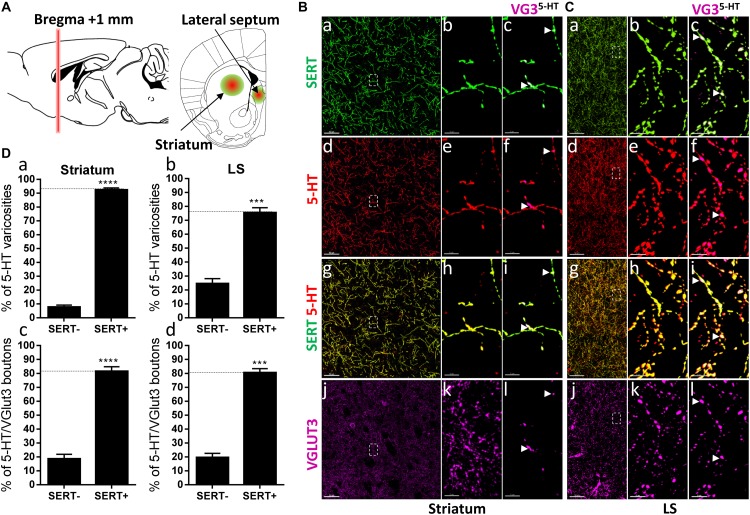
Distribution of VGLUT3^+^ boutons within 5-HT^+^ and SERT^+^ axons in the striatum and lateral septum. **(A)** Schematic drawing showing the location of the acquired micrographs. Mediolateral dorsal striatum and lateral septum (LS) mosaic images were acquired at bregma +1 ± 0.2 mm (red vertical line) (green/red circles). **(B)** Micrograph showing the distribution of the SERT (green, **a–c**), 5-HT (red, **d–f**), SERT (green) + 5-HT (red) **(g–i)**, and VGLUT3 (magenta, **j–l**) in the striatum. Left panel shows lower magnification. Scale bar: 50 μm. Middle **(b,e,h,k)** and right **(c,f,i,l)** panels show higher magnification of the white dashed box in the left panel **(a,d,g,j)**. Scale bar: 2 μm. The right panel shows the VGLUT3 boutons co-labeled with SERT **(c)**, 5-HT **(f)** and SERT + 5-HT **(i)**. VGLUT3 puncta located within 5-HT varicosities were isolated using Imaris **(l)**. Arrowheads show 5-HT varicosities collocated with SERT and VGLUT3 **(c,f,i,l)**. **(C)** Micrograph showing the distribution of the SERT (green, **a–c**), 5-HT (red, **d–f**), SERT (green) + 5-HT (red) **(g–i)**, and VGLUT3 (magenta, **j–l**) in the LS. Left panel shows lower magnification. Scale bar: 50 μm. Middle **(b,e,h,k)** and right **(c,f,i,l)** panels show higher magnification of the white dashed box in the left panel **(a,d,g,j)**. Scale bar: 2 μm. The right panel **(c,f,i,l)** shows the VGLUT3 boutons co-labeled with SERT **(c)**, 5-HT **(f)** and SERT + 5-HT **(i)**. VGLUT3 puncta located within 5-HT varicosities **(l)**. Arrowheads show 5-HT varicosities collocated with SERT and VGLUT3 **(c,f,i,l)**. **(D: a,b)** Quantification of the proportion of 5-HT varicosities not co-labeled (SERT^-^) and co-labeled (SERT^+^) with SERT in the striatum **(a)** and LS **(b)**, showing a great majority of 5-HT varicosities co-labeled with SERT. **(c,d)** Quantification of the proportion of 5-HT^+^/VGLUT3^+^ not co-labeled (SERT^-^) and co-labeled (SERT^+^) with SERT in the striatum **(c)** and LS **(d)**, showing a great majority of 5-HT^+^/VGLUT3^+^ varicosities co-labeled with SERT (*t*-test, ^∗∗∗∗^*p* < 0.0001; ^∗∗∗^*p* < 0.001).

**FIGURE 5:**
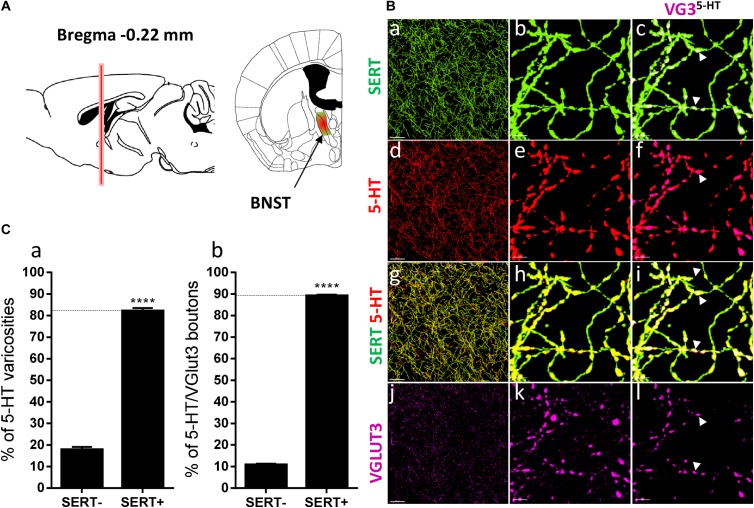
Distribution of VGLUT3^+^ boutons within 5-HT^+^ and SERT^+^ axons in the bed nucleus of the stria terminalis. **(A)** Schematic drawing showing the location of the acquired micrographs. The bed nucleus of the stria terminalis (BNST) mosaic images were acquired at bregma –0.22 ± 0.3 mm (red vertical line) in the medial posteromedial BNST (BSTMPM) and postero-intermediate part of the BNST (BSTMPI) (green/red area). **(B)** Micrograph showing the distribution of the SERT (green, **a–c**), 5-HT (red, **d–f**), SERT (green) + 5-HT (red) (**g–i**), and VGLUT3 (magenta, **j–l**) in the BNST. Left panel **(a,d,g,j)** shows lower magnification. Scale bar: 50 μm. Middle **(b,e,h,k)** and right **(c,f,i,l)** panels show higher magnification of the white dashed box in the left panel **(a,d,g,j)**. Scale bar: 2 μm. The right panel **(c,f,i,l)** shows the VGLUT3 boutons co-labeled with SERT **(c)**, 5-HT **(f)**, and SERT + 5-HT **(i)**. VGLUT3 puncta located within 5-HT varicosities were isolated using Imaris **(l)**. Arrowheads show 5-HT varicosities collocated with SERT and VGLUT3 **(c,f,i,l)**. **(C:a)** Quantification of the proportion of 5-HT varicosities not co-labeled (SERT^-^) and co-labeled (SERT^+^) with SERT in the BNST, showing a great majority of 5-HT varicosities co-labeled with SERT. **(b)** Quantification of the proportion of 5-HT^+^/VGLUT3^+^ not co-labeled (SERT^-^) and co-labeled (SERT^+^) with SERT in the BNST, showing a great majority of 5-HT^+^/VGLUT3^+^ varicosities co-labeled with SERT (*t*-test, ^∗∗∗∗^*p* < 0.0001).

**FIGURE 6:**
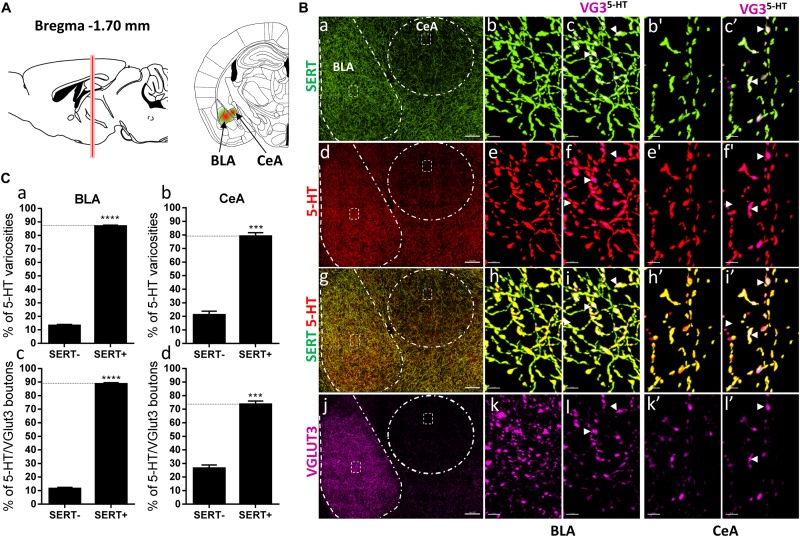
Distribution of VGLUT3^+^ boutons within 5-HT^+^ and SERT^+^ axons in the basolateral amygdala and central nucleus of the amygdala. **(A)** Schematic drawing showing the location of the acquired micrographs. The basolateral amygdala (BLA) and central nucleus of the amygdala (CeA) mosaic images were acquired at bregma –1.70 ± 0.2 mm (red vertical line) in the anterior part of the basolateral amygdala and the lateral CeA (green/red areas). **(B)** Micrograph showing the distribution of the SERT (green, **a–c,b’,c’**), 5-HT (red, **d–f,e’,f’**), SERT (green) + 5-HT (red) **(g–i,h’,i’)**, and VGLUT3 (magenta, **j–l,k’,l’**) in the CeA and BLA. Left panel **(a,d,g,j)** shows lower magnification. Scale bar: 150 μm. Middle **(b,e,h,k and b’,e’,h’,k’)** and right **(c,f,i,l and c’,i’,f’,l’)** panels show higher magnification of the white dashed areas in the left panel **(a,d,g,j)**. Scale bar: 2 μm. The right panels **(c,f,i,l and c’,f’,i’,l’)** shows the VGLUT3 boutons co-labeled with SERT **(c,c’)**, 5-HT **(f,f’)**, and SERT + 5-HT **(i,i’)** in the BLA and CeA. VGLUT3 puncta located within 5-HT varicosities were isolated using Imaris **(l,l’)**. Arrowheads show 5-HT varicosities collocated with SERT and VGLUT3 **(c,f,i,l and c’,f’,i’,l’)**. **(C: a,b)** Quantification of the proportion of 5-HT varicosities not co-labeled (SERT^-^) and co-labeled (SERT^+^) with SERT in the BLA **(a)** and CeA **(b)**, showing a great majority of 5-HT varicosities co-labeled with SERT. **(c,d)** Quantification of the proportion of 5-HT^+^/VGLUT3^+^ not co-labeled (SERT–) and co-labeled (SERT^+^) with SERT in the BLA **(c)** and CeA **(d)**, showing a great majority of 5-HT^+^/VGLUT3^+^ varicosities co-labeled with SERT (*t*-test, ^∗∗∗∗^*p* < 0.0001; ^∗∗∗^*p* < 0.001).

**FIGURE 7:**
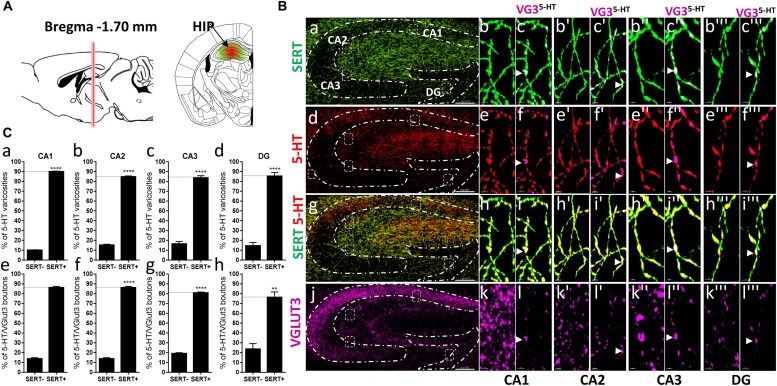
Distribution of VGLUT3^+^ boutons within 5-HT^+^ and SERT^+^ axons in the CA1, CA2, CA3, dentate gyrus of the hippocampus. **(A)** Schematic drawing showing the location of the acquired micrographs. The hippocampus (HIP) mosaic images were acquired at bregma –1.70 ± 0.3 mm (red vertical line) in the dorsal hippocampus (green/red area). **(B)** Micrograph showing the distribution of the SERT (green, **a–c”’**), 5-HT (red, **d–f”’**), SERT (green) + 5-HT (red) (**g–i”’**), and VGLUT3 (magenta, **j–l”’**) in the CA1, CA2, CA3, and DG. Left panel **(a,d,g,j)** shows lower magnification. Scale bar: 150 μm. Right panels show higher magnification of the CA1 **(b–l)**, CA2 **(b’–l’)**, CA3 **(b”–l”)**, and DG **(b”’–l”’)** in the white dashed box in the left panel **(a,d,g,j)**. Scale bar: 2 μm. The right panel shows the VGLUT3 boutons co-labeled with SERT **(c–c”’)**, 5-HT **(f–f”’)**, and SERT + 5-HT **(i–i”’)**. VGLUT3 puncta located within 5-HT varicosities were isolated using Imaris in the CA1 **(l–l”’)**. Arrowheads show 5-HT varicosities collocated with SERT and VGLUT3 in the CA1 (**c–c”’, f–f”’, i–i”’, and l–l”’**). **(C:a–d)** Quantification of the proportion of 5-HT varicosities not co-labeled (SERT^-^) and co-labeled (SERT^+^) with SERT in the CA1 **(a)**, CA2 **(b)**, CA3 **(c)**, and DG **(d)** of the HIP, showing a great majority of 5-HT varicosities co-labeled with SERT. **(e–h)** Quantification of the proportion of 5-HT + /VGLUT3+ not co-labeled (SERT^-^) and co-labeled (SERT^+^) with SERT in the CA1 **(e)**, CA2 **(f)**, CA3 **(g)**, and DG **(h)** of the HIP, showing a great majority of 5-HT^+^/VGLUT3^+^ varicosities co-labeled with SERT (*t*-test, ^∗∗∗∗^*p* < 0.0001; ^∗∗^*p* < 0.01).

Sections (3 sections per animal, *n* = 6 animals, 18 sections/brain region) were imaged on an Olympus FV3000 using a 60X oil-immersion objective (NA 1.35) with a 2.5x zoom and a *z*-axis step of 0.3 μm, using sequential scanning. Mosaics of the regions of interest were acquired as depicted in A of Figure 1–7, in OIR file format. The 5-HT immunoreactive boutons were reconstructed in 3D using the surface rendering function with Imaris 9.2.1 (Bitplane), as previously described (Belmer et al., 2016; Tarren et al., 2017). All the images were processed in batch using the same surface thresholding parameters. Mean fluorescence intensities of SERT or VGLUT3 labeling within 5-HT boutons and image volumes were obtained from the surface statistics in Imaris. Since the level of background of confocal images can reach as much as 30% of maximum image intensity (Landmann and Marbet, 2004), we use this threshold as a criteria to define the 5-HT boutons colocalized and non-colocalized with SERT and/or VGLUT3 (i.e., mean intensity >30% or < 30% of the maximum intensity, respectively). For each brain region, the frequency distribution of the colocalized and non-colocalized boutons within each brain region was analyzed using Excel 365, averaged for each animal, and plotted in Graphpad Prism 7.0 (Graph Pad Software Co., San Diego, CA, United States) as replicates (*n* = 6). Quantification of the degree of colocalization between the different channels in selected brain regions using Mander’s overlap coefficients was performed using Coloc2 plugin in Image J/Fiji (NIH). 3D representation of the proportion of VGLUT3 immunoreactive 5-HT boutons within each brain region was generated using the online Scalable Brain Atlas (Bakker et al., 2015) with a custom color-coded scale (Figure 9).

### Statistics

Statistical analyses were carried out using GraphPad Prism 7.0. The proportion of 5-HT or 5-HT/VGLUT3 varicosities immunopositive for SERT were compared to SERT immunonegative boutons within each brain region using a two-tailed unpaired *t*-test. The densities and relative densities of 5-HT/VGLUT3 boutons within all the analyzed brain regions were compared using one-way ANOVA with Bonferroni correction for multiple comparison. Correlation analysis of the density of 5-HT^+^/VGLUT3^+^ varicosities and density of 5-HT was performed using the Pearson correlation test. A *p*-value <0.05 was considered significant, with all values expressed as the mean ± SEM.

## Results

Previous studies have suggested that VGLUT3 always segregates with SERT within the varicosities of the 5-HT axons projecting to the prelimbic region of the prefrontal cortex (Amilhon et al., 2010). Therefore, we first examined the distribution of VGLUT3 and SERT immunoreactive 5-HT varicosities within the different layers of the prelimbic cortex (PrL), at bregma +2.46 mm (Figure 1A). Co-labeling of the SERT and 5-HT revealed that most of the varicosities reconstructed were co-labeled for the two markers (Figure 1B: a–i’). Labeling of VGLUT3 showed the typical scattered punctate fluorescence (Figure 1B: j) and by combining the 3D-reconstruction and the masking functions of Imaris, we isolated the VGLUT3 labeling that was only contained in 3D-reconstructed 5-HT varicosities (Figure 1B: k,k’). We observed that almost all the 5-HT/VGLUT3 boutons were co-labeled with SERT (arrowheads, Figure 1B: c,f,i,l and c’,f’,i’,l’). Indeed, the absolute quantifications of the proportion of 5-HT varicosities that were co-labeled with SERT confirmed that 85 and 91% of 5-HT varicosities were co-labeled with SERT in the layers 4–5 (*t* = 17.64, *df* = 8, *p* < 0.0001) and 1–3 (*t* = 32.01, *df* = 8, *p* < 0.0001), respectively (Figure 1C: a,b). Similar proportions of 5-HT/VGLUT3 boutons (82%, *t* = 11.12, df = 8, *p* < 0.0001 and 91%, *t* = 29.19, *df* = 8, *p* < 0.0001) were also co-labeled with SERT (Figure 1C: c,d). These results suggest that only a small proportion of 5-HT boutons (9–18%) do not express detectable levels of SERT in the PrL and, that VGLUT3 and SERT do not predominantly segregate within the 5-HT axonal varicosities in this brain region.

The co-release of 5-HT and glutamate by 5-HT neurons from the dorsal raphe has been proposed to play an important role in the regulation of the neurotransmission in the ventral striatum and the modulation of reward-related behaviors (Liu et al., 2014). Hence, we next investigated the distribution of SERT and VGLUT3 immunolabeled varicosities within the NAc core and shell, at bregma +1.42 mm (Figure 2A). Both in the core (94%, *t* = 18.99, *df* = 9, *p* < 0.0001) and the shell (95%, *t* = 19.52, *df* = 2, *p* < 0.01) regions of the NAc, the great majority of 5-HT boutons were also immunoreactive for SERT (Figure 2B,C: a–i, D: a,b). Hence, a great proportion of 5-HT^+^/VGLUT3^+^ boutons were co-labeled with the SERT, both in the core (92%, *t* = 11.88, *df* = 8, *p* < 0.0001) and the shell (89%, *t* = 17.1, *df* = 10, *p* < 0.0001) (arrowheads, Figure 2B,C: c,f,i,l, D: c,d). These results support a high degree of overlap between 5-HT and SERT immunoreactive axons in the core and the shell of the rostral NAc (Brown and Molliver, 2000), and further highlight the absence of any particular segregation between SERT and VGLUT3 within 5-HT varicosities.

Brown and Molliver (2000) also observed that a subset of 5-HT axons lack SERT in the caudal part of the rat NAc shell. Therefore, we investigated the distribution of SERT and VGLUT3 within the 5-HT varicosities in the posterior NAc shell, at bregma +1.00 mm, at a similar level to the aforementioned study in rat (i.e., “septal pole” or “cone region”) (Figure 3A). Interestingly, we found the 5-HT varicosities either co-labeled (arrowheads) or not labeled (or only weakly/partially labeled, arrows) for the SERT in the posterior NAc shell (Figure 3B: a–i) and both SERT+ and SERT- serotonergic varicosities were co-labeled for VGLUT3 (Figure 3B: c,f,i–l). The quantification revealed that half of the 5-HT varicosities do not express the SERT in the posterior shell of the mouse NAc (*t* = 0.03096, *df* = 6; *p* = 0.97; Figure 3C: a) as previously described in rats. Although VGLUT3 was observed in some SERT-varicosities, this subtype of serotonergic boutons only represents a minority (11%), as the great majority (89%, *t* = 17.1, *df* = 10, Figure 3C: b) of the 5-HT^+^/VGLUT3^+^ boutons were also co-labeled for SERT. These results further suggest that VGLUT3 are rather colocalized than segregated with the SERT in 5-HT varicosities.

The segregation of SERT and VGLUT3 was also reported in the dorsal striatum and the LS (Voisin et al., 2016). Hence, we investigated their distribution at bregma +1.00 mm (Figure 4A). Again, the great majority of the 5-HT varicosities express the SERT in both the dorsal striatum (92%, *t* = 28.02, *df* = 11; Figure 4B: a–i, D: a) and LS (75%, *t* = 10.11, *df* = 5, Figure 4C: a–i, D: b). Therefore, no axonal segregation could be observed, indeed more than 80% of the VGLUT3 immunoreactive 5-HT boutons were also co-labeled for the SERT, in both the striatum and LS (Figure 4B,C: c,f,i,l, arrowheads; Figure 4D: c,d). These results further evidence that SERT and VGLUT3 cannot be used as markers to classify different 5-HTergic axonal subtypes.

DR 5-HT neurons that send VGLUT3 immunoreactive axons to the NAc also send collaterals to different brain regions, including the BNST, CeA, and BLA. We therefore investigated the distribution of SERT and VGLUT3 within 5-HT varicosities in those brain regions (Figure 5, 6). In the anterior BNST at bregma -0.22 mm (Figure 5A), we again observed a high degree of overlapping between SERT and 5-HT immunoreactivity (82%, *t* = 23.17, *df* = 17, *p* < 0.0001; Figure 5B: a–i, C: a), with a high proportion (89%, *t* = 71.51, *df* = 17, *p* < 0.0001) of 5-HT/VGLUT3 varicosities that were co-labeled for SERT (Figure 5B: a,f,i,l, arrowheads, C: b). Similarly, in the amygdala at bregma -1.70 mm (Figure 6A), the great majority of 5-HT boutons were co-labeled for SERT in both the BLA (87%, *t* = 27.6, *df* = 11, *p* < 0.0001) and CeA (79%, *t* = 16.58, *df* = 5, *p* < 0.0001; Figure 6B: a–i’, C: a,b). Consequently, a great proportion of 5-HT^+/^VGLUT3^+^ boutons were co-labeled with the SERT within the BLA (89%, *t* = 38.6 *df* = 11, *p* < 0.0001) and CeA (73%, *t* = 9.46, *df* = 5, *p* = 0.0002) (arrowheads, Figure 6B: i–l’, C: c,d).

In the hippocampus, VGLUT3 was shown to modulate 5-HTergic tone, and to stimulate VMAT2-dependent accumulation of 5-HT in synaptic vesicles (Amilhon et al., 2010), which further suggests that VGLUT3/5-HT synaptic cross-talk may play an important role in hippocampal-mediated behaviors such as anxiety and depression. Since these behaviors are also dependent upon SERT activity/blockade by serotonergic antidepressant, whether VGLUT3 and SERT segregate or colocalize within 5-HT varicosities in the hippocampus is question of great interest and could further help in developing improved therapeutics for the treatment of anxiety- or depression-related disorders. Therefore, we rigorously investigated the distribution of SERT and VGLUT3 in 5-HT axonal varicosities within the different subregions of the hippocampus, at bregma -1.70 ± 0.3 mm (Figure 7A). Most of 5-HT boutons were also immunoreactive for SERT in all four regions of the hippocampus, CA1 (90%, *t* = 87.01, *df* = 3, *p* < 0.0001), CA2 (85%, *t* = 45.56, *df* = 3, *p* < 0.0001), CA3 (84%, *t* = 14.07, *df* = 3, *p* = 0.0008), and DG (86%, *t* = 10.17, *df* = 3, *p* = 0.002) (Figure 7B: g–i”’, C: a–d). A similar proportion of 5-HT^+/^VGLUT3^+^ boutons were co-labeled with SERT in the CA1 (87%, *t* = 32.76, *df* = 3, *p* < 0.0001), CA2 (87%, *t* = 32.76, *df* = 3, *p* < 0.0001), CA3 (82%, *t* = 37.55, *df* = 3, *p* < 0.0001), and DG (78%, *t* = 4.766 *df* = 3, *p* = 0.0175) (arrowheads, Figure 7B: c–c”’,f–f”’,i–i”’,l–l”’, C: e–h).

The absence of segregation we observed was further confirmed by quantification of the degree of colocalization between the different fluorophores/markers in selected brain regions (DLS, LS, and dorsal HIP) using Mander’s overlap coefficients (MOC; Supplementary Figure S1).

**FIGURE 8:**
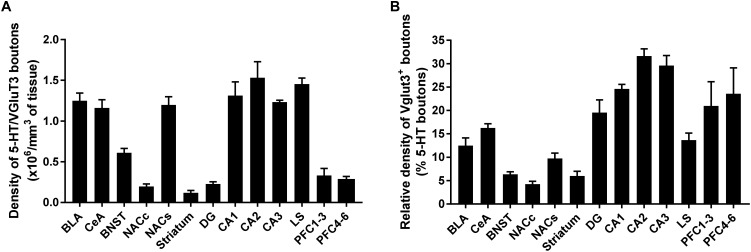
Quantification of 5-HT neurons expressing vesicular glutamate transporter (VGLUT3) in various regions of the mouse forebrain. Volumetric quantification of the density of VGLUT3 immunoreactive boutons within 5-HT-labeled fibers in each brain region. The results are expressed as density of boutons per 10^6^mm^3^ of 5-HT^+^ fiber **(A)**, or percent of boutons in 5-HT^+^ fiber **(B)** and represented as the mean ± SEM.

**FIGURE 9:**
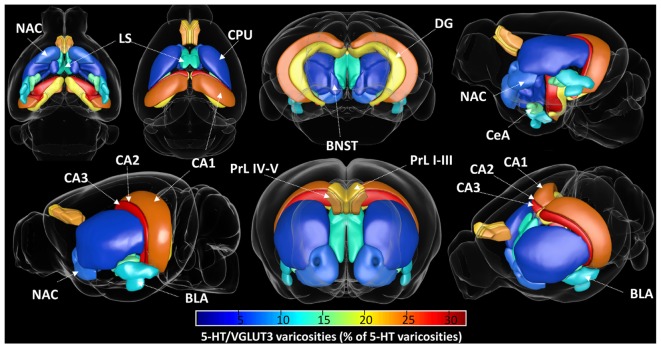
Visual representation of the proportion of 5-HT varicosities expressing the vesicular glutamate transporter VGLUT3 in various regions of the mouse forebrain. The percentage of total 5-HT varicosities that are co-labeled with VGLUT3 is represented with a color coding from 0 (dark blue) to 32% (dark red) within each analyzed brain region, from various viewing angle. The highest proportion of VGLUT3 was found in CA2, followed by CA3, CA1, PrL IV-V, PrL I-III, DG, CeA, BLA, NAc shell, BNST, and NAc core.

We have reconstructed a total of 10^6^ serotonergic varicosities from various brain regions, including the prelimbic cortex (5 × 10^4^), NAc (10 × 10^4^), LS (15 × 10^4^), BNST (25 × 10^4^), amygdala (27 × 10^4^), hippocampus (15 × 10^4^), and dorsal striatum (5 × 10^4^). Our quantification of the volumetric density of 5-HT^+^/VGLUT3^+^ boutons revealed a high heterogeneity along the different brain regions. The, CA1–3, LS, amygdala (BLA and CeA) and NAc shell show the highest density, and the NAc core, striatum, DG and prelimbic cortex show the lowest density of 5-HT^+^/VGLUT3^+^ varicosities [Figure 8A, One-way ANOVA, *F*_(12,94)_ = 10.55, *p* < 0.0001 see Supplementary Table 1 for Bonferroni’s multiple comparisons]. The relative density of 5-HT^+^/VGLUT3^+^ boutons, calculated as a percentage of total 5-HT varicosities within each brain region, shows the largest proportion is located in CA1–3 (24–32%), followed by the PrL (20–23%) > DG (19%) > CeA (16%) > LS (14%) > BLA (13%) > NAc shell (10%) > BNST (7%) > striatum (6%) > NAc core (4.2%) [Figure 8B, *F*_(12,94)_ = 46.37, *p* < 0.0001, see Supplementary Table 2 for Bonferroni’s multiple comparisons, and Figure 9]. Correlation analysis shows that the density of 5-HT^+^/VGLUT3^+^ varicosities is totally independent of the density of 5-HT boutons within each brain region (Pearson’s coefficients *r* = 0.32 and *R*^2^ = 0.10, *p* = 0.28). This data suggests that the heterogeneous distribution of 5-HT^+^/VGLUT3^+^ varicosities in the forebrain represents a functionally relevant feature of 5-HT neurons complex topology, rather than a biased detection of those varicosities in our methodological approach.

## Discussion

The main finding of the present study is that SERT and VGLUT3 rarely segregate within 5-HT varicosities, but rather preferentially colocalize in most of the mouse forebrain regions we analyzed. These results are in agreement with a previous observation in rats (Gagnon and Parent, 2014), but surprisingly differ from two previous studies in WT littermates (vglut3^+/+^) of a transgenic mouse line, obtained by the breeding of vglut3^+/-^ mice (Amilhon et al., 2010; Voisin et al., 2016). In these mice with mixed C57Bl/6 and 129/Sv backgrounds, a clear segregation of SERT and VGLUT3 was reported in the varicosities of the 5-HTergic axons projecting to the prelimbic cortex, the ventral and dorsal CA3 field of the hippocampus, the dorsal striatum and the LS (Amilhon et al., 2010; Voisin et al., 2016). In our study in pure wild-type C57Bl6/j background male and female mice of similar age (around P60), we did not observe this segregation between SERT and VGLUT3 in any brain region, suggesting that this difference may originate from mouse strain or gender variations. These discrepancies could also arise from differences in the methodology, probably related to antibody specificity or sensitivity. Indeed, the goat-anti SERT antibody used by Voisin et al. (2016; Santa Cruz; C-20) in their quantification was shown to yield non-specific binding on brain extracts from SERT knock-out mice, as opposed to the rabbit anti-SERT antibody from Calbiochem (PC177) used in the present study which was shown to be highly specific (Biezonski and Meyer, 2010). Further, Voisin et al. (2016) performed their immunohistochemistry experiments on 100 μm-thick sections while we used 30 μm sections in the present study, which could also produce variations in antibody penetration and immunodetection. Furthermore, in their immunogold staining of SERT and VGLUT3, they reported the use of normal goat serum for blocking, in combination with anti-goat secondary antibodies to label the SERT. This could result in anti-goat antibodies binding non-specifically to goat serum IgGs bound to the sections, and lead to underestimation of SERT immunoreactivity. A comparison between the two antibodies would indeed be of high interest to identify the proposed discrepancies, however, the C-20 antibody from Santa Cruz has now been discontinued.

Further studies using comprehensive titrations of different commercially available antibodies, or using conditional co-expression of tagged/fluorescent VGLUT3 and SERT proteins selectively in 5-HT neurons such as TPH2-CRE mice, are therefore needed to confirm the colocalization or segregation of SERT and VGLUT3 within 5-HT axonal varicosities in the mouse brain.

Axonal segregation between SERT and VGLUT3 within 5-HT varicosities would imply that a significant subset of 5-HT varicosities do not express the SERT. However, our results demonstrate that the great majority of 5-HT boutons (about 90%) are immunoreactive for the SERT, in almost all the brain regions studied. This absence of segregation of SERT and VGLUT3 therefore argues against the existence of a subset of 5-HT terminals with enhanced extracellular 5-HT levels after release due to weak 5-HT reuptake (Voisin et al., 2016). The only brain region with an equivalent proportion of SERT-positive and SERT-negative 5-HT boutons was the posterior part of the NAc shell. This is in line with previous reports in rats showing that only subtle differences could be detected between 5-HT and of SERT immunostaining in particular brain regions, with only a subset of 5-HT axonal projections that lack the SERT in the posterior part of the NAc (Brown and Molliver, 2000). While we observed that only half of 5-HT varicosities express the SERT in this brain region, we found VGLUT3 preferentially localized within SERT immunoreactive varicosities with only a small proportion (10%) of VGLUT3 localized in boutons lacking the SERT. This further suggests that the ability to co-release 5-HT and glutamate from the same vesicles relies on the presence of effective 5-HT reuptake machinery in the varicosities.

While the existence of 5-HT immunoreactive axons lacking the SERT has been evidenced almost 20 years ago (Brown and Molliver, 2000), only little is known regarding the origin or physiology of those SERT^-^/5-HT^+^ axons. Since 5-HT could also be stored in dopaminergic neurons of the substantia nigra or the ventral tegmental area (Zhou et al., 2005), we assessed the potential dopaminergic nature of the SERT^-^/5-HT^+^ varicosities. The absence of immunoreactivity for the tyrosine hydroxylase confirmed the non-catecholaminergic phenotype of these varicosities (data not shown), however, we cannot rule out the possibility that these 5-HT^+^/SERT^-^ axons are not truly serotonergic. Retrograde tracing studies are currently performed in our laboratory to determine the nature and origin of this particular subset of SERT^-^/5-HT^+^ axons, i.e., whether they expressed all these projections originate from the dorsal, median, or caudal raphe, whether this particular subtype of 5-HT neurons are restricted to specific raphe subnuclei.

Our results also show that 5-HT axons differentially express VGLUT3 in various brain regions, with a relative density of 5-HT varicosities expressing VGLUT3 ranging from 5 to 10% (dorsal and ventral striatum, BNST) to 20–30% (hippocampus and prefrontal cortex) of total 5-HT varicosities. This suggests that glutamate co-release could be differentially involved in 5-HT signaling across brain regions. The high density of 5-HT/glutamate co-release sites that we identified in particular brain regions known to be involved in the control of emotion, reward and decision-making such as the amygdala, NAc shell, hippocampus and prefrontal cortex, evokes new potential mechanisms for the control of neuroplasticity in these brain regions, and its dysregulation in the development of addictive disorders (Kauer and Malenka, 2007). In line with this, genetic ablation of *vglut3* in mice was shown to predispose to cocaine abuse (Sakae et al., 2015), however, the authors reported that this mechanism is likely independent of 5-HT signaling but rather linked to an increased dopamine and glutamate signaling in the NAc, from VTA dopaminergic and cortical glutamatergic inputs, respectively. Since our results highlight a high density of 5-HT/VGLUT3 boutons in the hippocampus and NAC, where 5-HT is known to play an important role in the regulation of glutamate and dopamine release at the level of axon terminals (Fink and Göthert, 2007), further studies are needed to identify the contribution of serotonin/glutamate co-signaling in addictive behaviors.

Indeed, recent evidence supports a significant role of 5-HT/glutamate co-transmission in both reward and emotion (Amilhon et al., 2010; Liu et al., 2014). Further, the activation of dopamine- and cAMP-regulated phosphoprotein of M(r) 32,000 (DARPP-32) by dopamine/glutamate co-transmission has been postulated to act as a molecular switch that control the reward pathway plasticity that mediates the behavioral sensitization to various drugs of abuse (Nairn et al., 2004; Valjent et al., 2005). DARPP-32 also appears to be involved in some biochemical and behavioral actions of 5-HT (Svenningsson et al., 2002), however, there is no evidence to date that serotonin-induced DARPP-32 activation is mediated by 5-HT/glutamate co-transmission. Although our results suggest that 5-HT and glutamate are co-released in the ventral and dorsal striatum where DARPP-32 is likely recruited following exposure to drugs of abuse, further studies are required to determine the precise role played by 5-HT and/or glutamate and their different receptors in DARPP-32 activation, the subsequent neuroplastic changes and the cognitive/emotional deficits produced by drugs of abuse. Furthermore, genetic ablation of VGLUT3 in mice (vglut3-/-) produces increased anxiety (Amilhon et al., 2010; Sakae et al., 2018), enhanced fear and altered stress axis regulation (Balázsfi et al., 2018), concomitant to the desensitization of 5-HT1A autoreceptors (Amilhon et al., 2010), suggesting a potential cross-regulation between 5-HT1A receptors and glutamate co-release. In line with this, unpublished evidence from our laboratory suggests that chronic treatment with drugs targeting the 5-HT1A receptors alter the density of 5-HT/VGLUT3 varicosities in specific brain region, including the hippocampus. Since the desensitization of 5-HT1A autoreceptors is essential for the antidepressant effect of selective serotonin reuptake inhibitors (SSRIs) (Popa et al., 2010), notably in the hippocampus, this further suggests that selective pharmacological ablation of VGLUT3 might represent a potential adjunct for antidepressant therapy.

In conclusion, despite an evident absence of segregation between VGLUT3 and SERT in 5-HT varicosities, our study confirms previous observations that 5-HT neurons and their axonal projections are heterogeneous in the rodent brain. The differences observed in the topological expression of VGLUT3 in 5-HT varicosities within various brain regions support the idea that 5-HT/glutamate co-release is a mechanism that is tightly regulated. Whether the co-release of glutamate and serotonin is involved in a particular type of neuroplasticity, mediates distinct behaviors or is implicated in specific neuropsychiatric disorders still need to be determined.

## Ethics Statement

This study was carried out in accordance with the recommendations of National Health and Medical Research Council (NHMRC) guidelines to promote the well-being of animals used for scientific purposes and the Australian code for the care and use of animals for scientific purposes. The protocol was approved by the Queensland University of Technology Animal Ethics Committee and the University of Queensland Animal Ethics Committee.

## Author Contributions

AB performed the IHC experiments. AB and KB acquired the confocal images. KB analyzed the images. AB, KB, and SB designed the experiments and analyzed the results. AJ, OP, and FS assisted with the data collection and figure formatting. AB and KB drafted the manuscript. AB, KB, AJ, OP, and SB revised the manuscript. All authors approved the final version of the manuscript.

## Conflict of Interest Statement

The authors declare that the research was conducted in the absence of any commercial or financial relationships that could be construed as a potential conflict of interest.

## Abbreviations

5-HT: 5-hydroxytryptamine
AMG: amygdala
BLA: basolateral amygdala
BNST: bed nucleus of the stria terminalis
CA1: 2, or 3, cornu ammonis 1, 2 or 3
CeA: central amygdala
CPU: caudate putamen
DG: dentate gyrus
HIP: hippocampus
LS: lateral septum
NAc: nucleus accumbens
NAc core: nucleus accumbens core
NAc shell: nucleus accumbens shell
PrL I–III: prelimbic cortex layer I to III
PrL IV–V: prelimbic cortex layer IV and V
SERT: serotonin transporter
VGLUT3/VG3: vesicular glutamate transporter 3
