# Supplementary material for: Axonal Non-segregation of the Vesicular Glutamate Transporter VGLUT3 Within Serotonergic Projections in the Mouse Forebrain
Source: Front Cell Neurosci. 2019 May 10;13:193. doi: 10.3389/fncel.2019.00193 (PMC6523995; doi:10.3389/fncel.2019.00193)
Supplement: Supplementary file 1 [file Table_1.DOCX]

**Table 1:** One-way ANOVA analysis of the volumetric density of 5-HT/VGLUT3 in the mouse forebrain. *(Significant changes are highlighted in light grey)*

| **F (12, 88) = 46.37** Bonferroni's multiple comparisons test | Mean Diff. | 95.00% CI of diff. | Significant | P Value | t |
| --- | --- | --- | --- | --- | --- |
|  |  |  |  |  |  |
| BLA vs. CeA | 0.08883 | -0.3068 to 0.4845 | ns | >0.9999 | 0.7949 |
| BLA vs. BNST | 0.6377 | 0.3283 to 0.9472 | **** | <0.0001 | 7.296 |
| BLA vs. NACc | 1.053 | 0.7081 to 1.398 | **** | <0.0001 | 10.81 |
| BLA vs. NACs | 0.0501 | -0.3282 to 0.4284 | ns | >0.9999 | 0.4688 |
| BLA vs. CPU | 1.131 | 0.7998 to 1.462 | **** | <0.0001 | 12.1 |
| BLA vs. CA1 | -0.06358 | -0.5147 to 0.3875 | ns | >0.9999 | 0.499 |
| BLA vs. CA2 | -0.2836 | -0.7347 to 0.1675 | ns | >0.9999 | 2.226 |
| BLA vs. CA3 | 0.01617 | -0.4349 to 0.4673 | ns | >0.9999 | 0.1269 |
| BLA vs. DG | 1.022 | 0.5708 to 1.473 | **** | <0.0001 | 8.02 |
| BLA vs. LS | -0.2063 | -0.602 to 0.1893 | ns | >0.9999 | 1.846 |
| BLA vs. PrL1-3 | 0.9156 | 0.5617 to 1.269 | **** | <0.0001 | 9.16 |
| BLA vs. PrL4-5 | 0.9743 | 0.6205 to 1.328 | **** | <0.0001 | 9.748 |
| CeA vs. BNST | 0.5489 | 0.1924 to 0.9054 | **** | <0.0001 | 5.452 |
| CeA vs. NACc | 0.9642 | 0.5765 to 1.352 | **** | <0.0001 | 8.806 |
| CeA vs. NACs | -0.03874 | -0.4564 to 0.3789 | ns | >0.9999 | 0.3284 |
| CeA vs. CPU | 1.042 | 0.6667 to 1.417 | **** | <0.0001 | 9.829 |
| CeA vs. CA1 | -0.1524 | -0.637 to 0.3321 | ns | >0.9999 | 1.114 |
| CeA vs. CA2 | -0.3724 | -0.857 to 0.1121 | ns | 0.6118 | 2.721 |
| CeA vs. CA3 | -0.07267 | -0.5572 to 0.4119 | ns | >0.9999 | 0.5309 |
| CeA vs. DG | 0.9331 | 0.4485 to 1.418 | **** | <0.0001 | 6.818 |
| CeA vs. LS | -0.2952 | -0.7286 to 0.1382 | ns | >0.9999 | 2.411 |
| CeA vs. PrL1-3 | 0.8267 | 0.4311 to 1.222 | **** | <0.0001 | 7.398 |
| CeA vs. PrL4-5 | 0.8855 | 0.4899 to 1.281 | **** | <0.0001 | 7.924 |
| BNST vs. NACc | 0.4153 | 0.1161 to 0.7145 | *** | 0.0003 | 4.915 |
| BNST vs. NACs | -0.5876 | -0.9248 to -0.2505 | **** | <0.0001 | 6.171 |
| BNST vs. CPU | 0.4931 | 0.2101 to 0.7762 | **** | <0.0001 | 6.168 |
| BNST vs. CA1 | -0.7013 | -1.118 to -0.2841 | **** | <0.0001 | 5.952 |
| BNST vs. CA2 | -0.9213 | -1.338 to -0.5041 | **** | <0.0001 | 7.819 |
| BNST vs. CA3 | -0.6216 | -1.039 to -0.2044 | **** | <0.0001 | 5.275 |
| BNST vs. DG | 0.3842 | -0.03298 to 0.8014 | ns | 0.1234 | 3.261 |
| BNST vs. LS | -0.8441 | -1.201 to -0.4876 | **** | <0.0001 | 8.383 |
| BNST vs. PrL1-3 | 0.2779 | -0.0316 to 0.5873 | ns | 0.1592 | 3.179 |
| BNST vs. PrL4-5 | 0.3366 | 0.02716 to 0.6461 | * | 0.0174 | 3.851 |
| NACc vs. NACs | -1.003 | -1.373 to -0.633 | **** | <0.0001 | 9.598 |
| NACc vs. CPU | 0.07784 | -0.2436 to 0.3993 | ns | >0.9999 | 0.8574 |
| NACc vs. CA1 | -1.117 | -1.561 to -0.6725 | **** | <0.0001 | 8.901 |
| NACc vs. CA2 | -1.337 | -1.781 to -0.8925 | **** | <0.0001 | 10.66 |
| NACc vs. CA3 | -1.037 | -1.481 to -0.5927 | **** | <0.0001 | 8.266 |
| NACc vs. DG | -0.0311 | -0.4752 to 0.413 | ns | >0.9999 | 0.2479 |
| NACc vs. LS | -1.259 | -1.647 to -0.8717 | **** | <0.0001 | 11.5 |
| NACc vs. PrL1-3 | -0.1374 | -0.4824 to 0.2075 | ns | >0.9999 | 1.411 |
| NACc vs. PrL4-5 | -0.07867 | -0.4236 to 0.2662 | ns | >0.9999 | 0.8075 |
| NACs vs. CPU | 1.081 | 0.7237 to 1.438 | **** | <0.0001 | 10.72 |
| NACs vs. CA1 | -0.1137 | -0.5842 to 0.3568 | ns | >0.9999 | 0.8554 |
| NACs vs. CA2 | -0.3337 | -0.8042 to 0.1368 | ns | >0.9999 | 2.511 |
| NACs vs. CA3 | -0.03393 | -0.5044 to 0.4366 | ns | >0.9999 | 0.2553 |
| NACs vs. DG | 0.9718 | 0.5013 to 1.442 | **** | <0.0001 | 7.313 |
| NACs vs. LS | -0.2564 | -0.6741 to 0.1612 | ns | >0.9999 | 2.174 |
| NACs vs. PrL1-3 | 0.8655 | 0.4872 to 1.244 | **** | <0.0001 | 8.1 |
| NACs vs. PrL4-5 | 0.9242 | 0.5459 to 1.303 | **** | <0.0001 | 8.65 |
| CPU vs. CA1 | -1.194 | -1.628 to -0.761 | **** | <0.0001 | 9.757 |
| CPU vs. CA2 | -1.414 | -1.848 to -0.981 | **** | <0.0001 | 11.55 |
| CPU vs. CA3 | -1.115 | -1.548 to -0.6813 | **** | <0.0001 | 9.106 |
| CPU vs. DG | -0.1089 | -0.5423 to 0.3245 | ns | >0.9999 | 0.8899 |
| CPU vs. LS | -1.337 | -1.713 to -0.9619 | **** | <0.0001 | 12.61 |
| CPU vs. PrL1-3 | -0.2153 | -0.5463 to 0.1157 | ns | >0.9999 | 2.303 |
| CPU vs. PrL4-5 | -0.1565 | -0.4875 to 0.1745 | ns | >0.9999 | 1.674 |
| CA1 vs. CA2 | -0.22 | -0.7508 to 0.3108 | ns | >0.9999 | 1.467 |
| CA1 vs. CA3 | 0.07975 | -0.4511 to 0.6106 | ns | >0.9999 | 0.5319 |
| CA1 vs. DG | 1.086 | 0.5547 to 1.616 | **** | <0.0001 | 7.24 |
| CA1 vs. LS | -0.1428 | -0.6273 to 0.3418 | ns | >0.9999 | 1.043 |
| CA1 vs. PrL1-3 | 0.9792 | 0.5281 to 1.43 | **** | <0.0001 | 7.685 |
| CA1 vs. PrL4-5 | 1.038 | 0.5868 to 1.489 | **** | <0.0001 | 8.146 |
| CA2 vs. CA3 | 0.2998 | -0.2311 to 0.8306 | ns | >0.9999 | 1.999 |
| CA2 vs. DG | 1.306 | 0.7747 to 1.836 | **** | <0.0001 | 8.707 |
| CA2 vs. LS | 0.07725 | -0.4073 to 0.5618 | ns | >0.9999 | 0.5644 |
| CA2 vs. PrL1-3 | 1.199 | 0.7481 to 1.65 | **** | <0.0001 | 9.411 |
| CA2 vs. PrL4-5 | 1.258 | 0.8068 to 1.709 | **** | <0.0001 | 9.873 |
| CA3 vs. DG | 1.006 | 0.4749 to 1.537 | **** | <0.0001 | 6.708 |
| CA3 vs. LS | -0.2225 | -0.7071 to 0.2621 | ns | >0.9999 | 1.626 |
| CA3 vs. PrL1-3 | 0.8994 | 0.4483 to 1.351 | **** | <0.0001 | 7.059 |
| CA3 vs. PrL4-5 | 0.9582 | 0.5071 to 1.409 | **** | <0.0001 | 7.52 |
| DG vs. LS | -1.228 | -1.713 to -0.7437 | **** | <0.0001 | 8.974 |
| DG vs. PrL1-3 | -0.1063 | -0.5574 to 0.3448 | ns | >0.9999 | 0.8346 |
| DG vs. PrL4-5 | -0.04757 | -0.4987 to 0.4035 | ns | >0.9999 | 0.3734 |
| LS vs. PrL1-3 | 1.122 | 0.7263 to 1.518 | **** | <0.0001 | 10.04 |
| LS vs. PrL4-5 | 1.181 | 0.785 to 1.576 | **** | <0.0001 | 10.57 |
| PrL1-3 vs. PrL4-5 | 0.05877 | -0.2951 to 0.4126 | ns | >0.9999 | 0.5879 |

**Table 2:** One-way ANOVA analysis of the relative density of 5-HT/VGLUT3 in the mouse forebrain. *(Significant changes are highlighted in light grey)*

| **F (12, 94) = 10.55** Bonferroni's multiple comparisons test | Mean Diff. | 95.00% CI of diff. | Significant | P Value | t |
| --- | --- | --- | --- | --- | --- |
|  |  |  |  |  |  |
| BLA vs. CeA | -3.801 | -16.89 to 9.285 | ns | >0.9999 | 1.026 |
| BLA vs. BNST | 6.116 | -3.879 to 16.11 | ns | >0.9999 | 2.161 |
| BLA vs. NACc | 8.216 | -2.991 to 19.42 | ns | 0.8688 | 2.59 |
| BLA vs. NACs | 2.695 | -8.231 to 13.62 | ns | >0.9999 | 0.8713 |
| BLA vs. CPU | 6.465 | -4.221 to 17.15 | ns | >0.9999 | 2.137 |
| BLA vs. CA1 | -12.17 | -27.28 to 2.946 | ns | 0.4272 | 2.843 |
| BLA vs. CA2 | -19.15 | -34.26 to -4.041 | ** | 0.0017 | 4.477 |
| BLA vs. CA3 | -17.12 | -32.23 to -2.007 | ** | 0.0098 | 4.001 |
| BLA vs. DG | -7.094 | -22.21 to 8.018 | ns | >0.9999 | 1.658 |
| BLA vs. LS | -1.215 | -14.3 to 11.87 | ns | >0.9999 | 0.3279 |
| BLA vs. PrL1-3 | -8.508 | -20.05 to 3.034 | ns | 0.8363 | 2.604 |
| BLA vs. PrL4-5 | -11.08 | -22.63 to 0.4574 | ns | 0.0793 | 3.392 |
| CeA vs. BNST | 9.918 | -2.612 to 22.45 | ns | 0.4897 | 2.796 |
| CeA vs. NACc | 12.02 | -1.498 to 25.53 | ns | 0.1759 | 3.141 |
| CeA vs. NACs | 6.497 | -6.787 to 19.78 | ns | >0.9999 | 1.727 |
| CeA vs. CPU | 10.27 | -2.821 to 23.35 | ns | 0.5258 | 2.771 |
| CeA vs. CA1 | -8.364 | -25.26 to 8.532 | ns | >0.9999 | 1.748 |
| CeA vs. CA2 | -15.35 | -32.25 to 1.544 | ns | 0.142 | 3.209 |
| CeA vs. CA3 | -13.32 | -30.21 to 3.579 | ns | 0.5064 | 2.784 |
| CeA vs. DG | -3.292 | -20.19 to 13.6 | ns | >0.9999 | 0.6883 |
| CeA vs. LS | 2.586 | -12.53 to 17.7 | ns | >0.9999 | 0.6045 |
| CeA vs. PrL1-3 | -4.706 | -18.5 to 9.089 | ns | >0.9999 | 1.205 |
| CeA vs. PrL4-5 | -7.283 | -21.08 to 6.512 | ns | >0.9999 | 1.865 |
| BNST vs. NACc | 2.1 | -8.451 to 12.65 | ns | >0.9999 | 0.703 |
| BNST vs. NACs | -3.421 | -13.67 to 6.83 | ns | >0.9999 | 1.179 |
| BNST vs. CPU | 0.3483 | -9.647 to 10.34 | ns | >0.9999 | 0.1231 |
| BNST vs. CA1 | -18.28 | -32.91 to -3.65 | ** | 0.0021 | 4.413 |
| BNST vs. CA2 | -25.27 | -39.9 to -10.64 | **** | <0.0001 | 6.1 |
| BNST vs. CA3 | -23.23 | -37.87 to -8.603 | **** | <0.0001 | 5.609 |
| BNST vs. DG | -13.21 | -27.84 to 1.422 | ns | 0.1514 | 3.189 |
| BNST vs. LS | -7.331 | -19.86 to 5.199 | ns | >0.9999 | 2.067 |
| BNST vs. PrL1-3 | -14.62 | -25.53 to -3.718 | *** | 0.0006 | 4.736 |
| BNST vs. PrL4-5 | -17.2 | -28.11 to -6.295 | **** | <0.0001 | 5.571 |
| NACc vs. NACs | -5.521 | -16.96 to 5.915 | ns | >0.9999 | 1.705 |
| NACc vs. CPU | -1.752 | -12.96 to 9.455 | ns | >0.9999 | 0.5521 |
| NACc vs. CA1 | -20.38 | -35.87 to -4.897 | *** | 0.0008 | 4.649 |
| NACc vs. CA2 | -27.37 | -42.85 to -11.88 | **** | <0.0001 | 6.243 |
| NACc vs. CA3 | -25.33 | -40.82 to -9.85 | **** | <0.0001 | 5.779 |
| NACc vs. DG | -15.31 | -30.79 to 0.1747 | ns | 0.0571 | 3.492 |
| NACc vs. LS | -9.431 | -22.95 to 4.085 | ns | >0.9999 | 2.465 |
| NACc vs. PrL1-3 | -16.72 | -28.75 to -4.698 | *** | 0.0003 | 4.912 |
| NACc vs. PrL4-5 | -19.3 | -31.33 to -7.274 | **** | <0.0001 | 5.669 |
| NACs vs. CPU | 3.77 | -7.156 to 14.7 | ns | >0.9999 | 1.219 |
| NACs vs. CA1 | -14.86 | -30.14 to 0.4222 | ns | 0.069 | 3.435 |
| NACs vs. CA2 | -21.85 | -37.13 to -6.565 | *** | 0.0002 | 5.049 |
| NACs vs. CA3 | -19.81 | -35.1 to -4.531 | ** | 0.0011 | 4.579 |
| NACs vs. DG | -9.789 | -25.07 to 5.494 | ns | >0.9999 | 2.262 |
| NACs vs. LS | -3.91 | -17.19 to 9.374 | ns | >0.9999 | 1.04 |
| NACs vs. PrL1-3 | -11.2 | -22.97 to 0.5616 | ns | 0.087 | 3.363 |
| NACs vs. PrL4-5 | -13.78 | -25.54 to -2.015 | ** | 0.006 | 4.137 |
| CPU vs. CA1 | -18.63 | -33.74 to -3.518 | ** | 0.0026 | 4.354 |
| CPU vs. CA2 | -25.62 | -40.73 to -10.51 | **** | <0.0001 | 5.988 |
| CPU vs. CA3 | -23.58 | -38.69 to -8.471 | **** | <0.0001 | 5.512 |
| CPU vs. DG | -13.56 | -28.67 to 1.553 | ns | 0.161 | 3.169 |
| CPU vs. LS | -7.68 | -20.77 to 5.407 | ns | >0.9999 | 2.073 |
| CPU vs. PrL1-3 | -14.97 | -26.51 to -3.431 | ** | 0.0011 | 4.582 |
| CPU vs. PrL4-5 | -17.55 | -29.09 to -6.007 | **** | <0.0001 | 5.37 |
| CA1 vs. CA2 | -6.988 | -25.5 to 11.52 | ns | >0.9999 | 1.334 |
| CA1 vs. CA3 | -4.953 | -23.46 to 13.55 | ns | >0.9999 | 0.9453 |
| CA1 vs. DG | 5.071 | -13.44 to 23.58 | ns | >0.9999 | 0.9678 |
| CA1 vs. LS | 10.95 | -5.945 to 27.85 | ns | >0.9999 | 2.289 |
| CA1 vs. PrL1-3 | 3.657 | -12.07 to 19.39 | ns | >0.9999 | 0.8213 |
| CA1 vs. PrL4-5 | 1.081 | -14.65 to 16.81 | ns | >0.9999 | 0.2427 |
| CA2 vs. CA3 | 2.034 | -16.47 to 20.54 | ns | >0.9999 | 0.3883 |
| CA2 vs. DG | 12.06 | -6.449 to 30.57 | ns | >0.9999 | 2.301 |
| CA2 vs. LS | 17.94 | 1.042 to 34.83 | * | 0.0238 | 3.75 |
| CA2 vs. PrL1-3 | 10.64 | -5.084 to 26.37 | ns | >0.9999 | 2.39 |
| CA2 vs. PrL4-5 | 8.068 | -7.66 to 23.8 | ns | >0.9999 | 1.812 |
| CA3 vs. DG | 10.02 | -8.483 to 28.53 | ns | >0.9999 | 1.913 |
| CA3 vs. LS | 15.9 | -0.9921 to 32.8 | ns | 0.0985 | 3.325 |
| CA3 vs. PrL1-3 | 8.61 | -7.118 to 24.34 | ns | >0.9999 | 1.934 |
| CA3 vs. PrL4-5 | 6.034 | -9.695 to 21.76 | ns | >0.9999 | 1.355 |
| DG vs. LS | 5.879 | -11.02 to 22.77 | ns | >0.9999 | 1.229 |
| DG vs. PrL1-3 | -1.414 | -17.14 to 14.31 | ns | >0.9999 | 0.3175 |
| DG vs. PrL4-5 | -3.991 | -19.72 to 11.74 | ns | >0.9999 | 0.8961 |
| LS vs. PrL1-3 | -7.293 | -21.09 to 6.502 | ns | >0.9999 | 1.867 |
| LS vs. PrL4-5 | -9.869 | -23.66 to 3.926 | ns | >0.9999 | 2.527 |
| PrL1-3 vs. PrL4-5 | -2.577 | -14.92 to 9.762 | ns | >0.9999 | 0.7376 |
