# Supplementary figures and images for: Axonal Non-segregation of the Vesicular Glutamate Transporter VGLUT3 Within Serotonergic Projections in the Mouse Forebrain
Source: Front Cell Neurosci. 2019 May 10;13:193. doi: 10.3389/fncel.2019.00193 (PMC6523995; doi:10.3389/fncel.2019.00193)

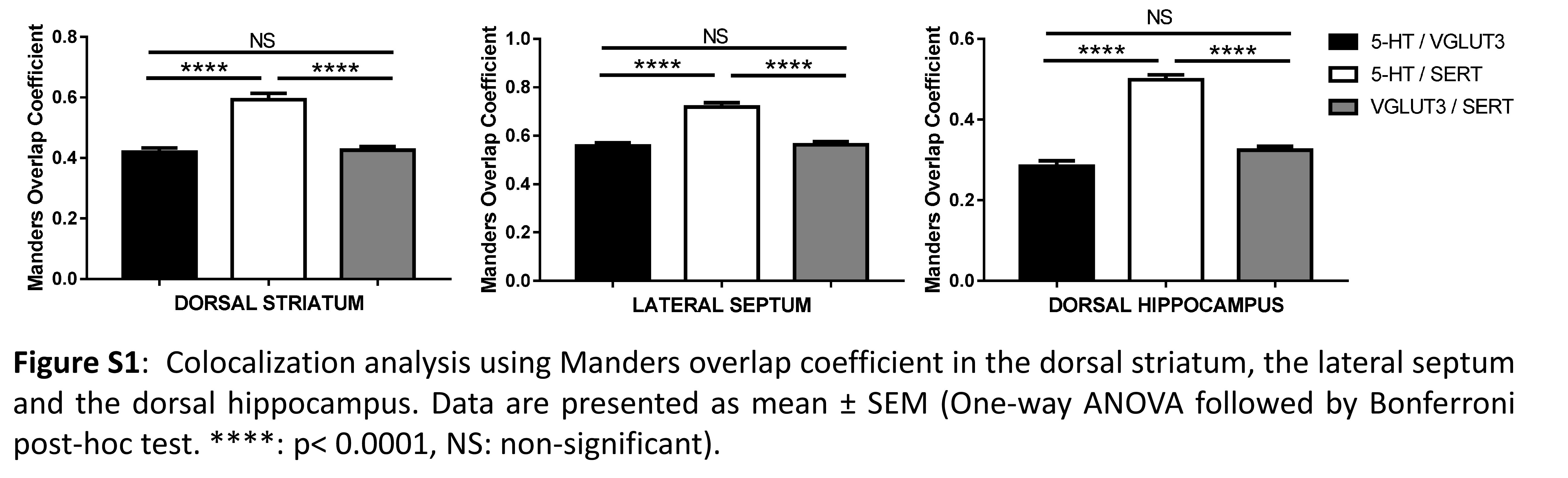

Supplement: Supplementary file 2 [file Image_1.tif]
